# Changes in knee pain and walking speed following primary, unilateral total knee arthroplasty and their association: A systematic review and meta-analysis

**DOI:** 10.1016/j.ocarto.2025.100694

**Published:** 2025-10-10

**Authors:** Nico Faber, Matej Skrobot, Georg N. Duda, Nicholas M. Brisson

**Affiliations:** Julius Wolff Institute, Berlin Institute of Health at Charité – Universitätsmedizin Berlin, Berlin, Germany

**Keywords:** Total knee arthroplasty, TKA, Knee pain, Walking speed, Systematic review and meta-analysis, Osteoarthritis

## Abstract

**Objective:**

To quantify changes in knee pain and walking speed following primary, unilateral total knee arthroplasty (TKA) for knee osteoarthritis, and to examine their relationship during recovery. Walking speed is a key indicator of functional recovery and long-term health, but whether pain relief translates into improved mobility remains unclear.

**Design:**

A systematic review and meta-analysis was conducted following PRISMA guidelines. PubMed was searched to September 2025 for studies reporting pre- and post-TKA values of knee pain (WOMAC-pain or KOOS-pain) and self-selected walking speed in adults undergoing primary, unilateral TKA. Pooled changes were calculated using inverse-variance weighted random-effects models. Meta-regression explored associations between pain and walking speed, adjusting for covariates.

**Results:**

Eighteen studies (n ​= ​819; 64.1 ​% female; age 65.1 ​± ​8.3 years; body mass index 28.4 ​± ​5.9 ​kg/m^2^) were included. Knee pain significantly decreased by 27–36 points (WOMAC-pain) and 19–40 points (KOOS-pain), exceeding minimal clinically important differences. Walking speed increased by +0.12 ​m/s at 3 months and +0.18 ​m/s at 12 months, both exceeding the +0.1 ​m/s threshold for clinically relevant change. No significant change in speed occurred before 3 months, and a non-significant decline appeared beyond 12 months. Meta-regression revealed greater knee pain (β ​= ​−0.005, p ​< ​0.001) and use of WOMAC-pain (vs. KOOS-pain) (β ​= ​−0.224, p ​< ​0.001) predicted slower walking speed (R^2^ ​= ​0.48, p ​< ​0.001).

**Conclusions:**

TKA yields substantial pain relief and improved walking speeds by 3–12 months. However, mobility gains are not sustained beyond one year and are influenced by pain. Postoperative care should support both symptoms and mobility to improve long-term outcomes.

## 1 Introduction

Osteoarthritis (OA) is the leading cause of chronic pain, mobility loss and functional disability – for which there is still no cure [1]. The knee is the most frequently affected joint, with a prevalence of 365 million knee OA cases worldwide in 2020 [2]. Total knee arthroplasty (TKA) is a leading treatment modality for addressing end-stage knee OA, promising improvements in pain, function and quality of life [3,4]. Perpetuated by an ageing global population and increasing rates of obesity [2,5], the rates of knee OA – and consequently TKA – are rising [2,6]. For instance, knee OA cases are projected to increase by ∼75 ​% from 2020 to 2050 globally [2], while TKA cases are projected to increase by ∼36 ​% from 2019 to 2060 in the USA [6]. These data underscore the urgent need for effective management and treatment strategies, and robust markers to evaluate their success.

Patient expectations are a crucial determining factor of TKA outcomes [7]. The three most frequently reported expectations among individuals undergoing TKA are pain reduction, the ability to resume daily activities, and improved mobility (i.e., walking ability) [8]. Due to the rise of TKA in younger patients and the rise in life expectancy (i.e., quality-adjusted life years), modern patients have higher expectations around their ability to return to recreational and sport activities [7,9,10]. These facts highlight the importance of TKA being able to effectively alleviate pain and restore mobility simultaneously to successfully enable patients to return to daily living, recreational and sport activities.

TKA is widely recognized as an effective treatment for knee OA, providing pain relief and improved function [4,7,[11], [12], [13]]. Although systematic reviews using standardized pain scales have reported that primary TKA generally reduces knee pain [4,11,12], important limitations restrict a comprehensive assessment of its pain-relieving impact. For instance, some reviews did not quantify preoperative pain [11], included small or mixed surgical cohorts [4], or failed to evaluate the magnitude of pre-to-postoperative pain reduction [12]. The meta-regression by Sayah et al. (2021) also did not account for potential covariates such as age, sex, or body mass index (BMI) [12]. Despite these insightful studies and the use of standardized pain metrics, no meta-analysis or meta-regression has yet objectively quantified pre-to-postoperative knee pain changes exclusively in primary, unilateral TKA patients.

The effect of TKA on functional mobility has also been investigated previously [14,15]. In particular, walking speed – sometimes considered the “sixth vital sign” for its reflection of both knee function and overall health – serves as a key measure of functional mobility [16,17]. Specifically, two meta-analyses have reported that knee joint replacement generally results in increased walking speed [14,15]. Nevertheless, these reviews did not exclusively examine patients with primary, unilateral TKA; instead, they included individuals with unicompartmental and bilateral arthroplasties and combined self-selected with fixed-speed assessments. Because functional outcomes can vary by joint replacement type [18,19], and fixed-speed (i.e., experimentally controlled) protocols fail to replicate the natural, repeatable mechanical environment of the knee joint during daily life – where arthritic disease developed [20] – these reviews may not accurately reflect the specific impact of unilateral TKA on everyday walking speed.

Walking speed is a critical clinical outcome following TKA. Slower walking speeds are associated with reduced functional capacity, poorer physical and cognitive health, and lower survival rates in older adults [17,21], while also indicating health risks in midlife adults [22,23]. Moreover, walking speed predicts rehabilitation response, functional dependence, frailty, and fall risk – outcomes particularly relevant to TKA patients [17]. Collectively, these findings highlight the broad clinical significance of walking speed. While pain relief is a key goal of TKA, its direct impact on walking speed remains unclear. Given the clinical importance of both, examining their relationship is essential.

Although reductions in knee pain are often assumed to improve physical function – given the strong association between pain and functional limitations [24,25], and the parallel improvements frequently observed with pain-targeted interventions [26] – this relationship is not guaranteed. In clinical settings, patients may experience significant pain relief after TKA without corresponding gains in walking speed [27]. Higher pain levels have been linked to slower walking speed early after unilateral TKA [28,29], while greater reductions in pain have been associated with larger gains in walking speed two years after bilateral TKA [30]. Therefore, it is critical to determine whether, and to what extent, knee pain is associated with walking speed in TKA patients. This underscores the importance of systematically reviewing how pain and walking speed change together.

This systematic review and meta-analysis aims to synthesize current evidence on the simultaneous changes in knee pain and walking speed following primary, unilateral TKA for knee OA. By aggregating data from multiple studies, we seek to provide a comprehensive understanding of both the symptomatic and functional benefits of TKA. Clarifying the relationship between pain and functional mobility will not only inform clinical practice and enhance patient management but also underscore its broader health implications for the TKA population.

## 2 Methods

This study conformed to the Preferred Reporting Items for Systematic Reviews and Meta-Analyses (PRISMA) methodological guidelines [31] and the Cochrane Handbook for Systematic Reviews of Interventions [32]. The review protocol was registered with the Open Science Framework (registration DOI: 10.17605/OSF.IO/83BAK).

### 2.1 Search strategy and eligibility criteria

PubMed was systematically searched through to September 30, 2025, using Medical Subject Headings (MeSH) and keyword combinations (Appendix 1). PubMed was selected for its comprehensive biomedical coverage [33]. Two authors (NF and MS) independently screened titles, abstracts, and full texts, resolving disagreements through discussion or third-party adjudication (NMB).

Studies were included if they evaluated: (i) individuals with primary, unilateral TKA; (ii) knee pain using the pain subscale of either the Western Ontario and McMaster Universities Osteoarthritis Index (WOMAC-pain) or the Knee Injury and Osteoarthritis Outcome Score (KOOS-pain); and (iii) self-selected walking speed. Studies had to report both pre-TKA and post-TKA values for pain and walking speed. The WOMAC-pain and KOOS-pain were selected for their validation and widespread use in knee OA and TKA populations, collectively appearing in more than 3300 studies [[34], [35], [36], [37], [38]]. All WOMAC-pain formats were included, as different versions demonstrate strong agreement [34,35,39,40].

Exclusion criteria were: (i) non-English studies; (ii) case reports; (iii) participants undergoing joint replacement surgery other than primary, unilateral TKA (e.g., unicompartmental arthroplasty, revision surgeries); and (iv) use of walking aids (e.g., canes, walkers) during speed assessments. Studies were not excluded based on age, sex and body mass index (BMI). Screening followed a predefined decision tree (Appendix 2). A supplementary search was conducted using Google Scholar with relevant terms, and reference lists from key reviews [4,11,12,14,15,[41], [42], [43]] were screened.

### 2.2 Data extraction

One author (NF) extracted data into a customized spreadsheet (Microsoft Excel, version 16.84), cross-checked by others (MS, NMB). Extracted data included: first author name and year of publication, sample size, sex, age, BMI, pain and speed measurement methods, inclusion or absence of a control group, timing of preoperative (PRE) and postoperative (POST) measurements, and pain and speed values at all reported time points. Categorical data were extracted as frequencies, and continuous data as means ​± ​standard deviations (SDs).

Unadjusted change-from-baseline means and SDs for knee pain and walking speed were extracted. If unavailable, study authors were contacted; in the absence of a response, reported values were converted as described below. When two closely spaced time points were reported, the one most aligned with other studies was selected.

WOMAC-pain scores were standardized to a 0–100 point scale using a commonly accepted method [34,40,44,45], where 0 represents no pain and 100 represents the worst pain:

$$Pain_{0-100}=\frac{Pain_{0-N}}{N}\times 100$$

where ***N*** is the maximum score achievable on the reported scale. If inverted WOMAC-pain scores were reported, values were adjusted by subtracting from 100. To align with the standardized WOMAC-pain scores, KOOS-pain values were inverted by subtracting each score from 100, so that higher scores uniformly reflect greater pain. Walking speed was standardized to meters per second (m/s). To avoid duplicate data, studies were checked for overlap in authorship, demographics, and outcomes. When unresolved, the earliest publication was used.

### 2.3 Risk of bias

Risk of bias was assessed using a modified Newcastle-Ottawa Scale (NOS) [46], adapted from Coburn et al. (2022) [47] to assess selection, measurement, and observation bias (Appendix 3). Two authors (NF, MS) independently assessed risk, with disagreements resolved by discussion or consultation (NMB). Studies in which more than half of the applicable items were scored as “low risk” were considered low risk overall. Funnel plots were generated to assess publication bias and heterogeneity using the General Package for Meta-Analysis (version 7.0.0) in R (version 4.3.3) [48].

### 2.4 Data synthesis and analysis

Pooled demographics were calculated using sample size-weighted means (Appendix 4A) [49]. If continuous data were not reported as means ​± ​SDs, they were estimated from medians, ranges, or confidence intervals (CIs) using established methods [50,51]. When multiple treatment arms were reported, data were aggregated per Cochrane guidelines [32]. The primary outcomes – changes in knee pain and walking speed from PRE to POST were calculated as the POST minus PRE when not directly reported. Missing change-from-baseline SDs were computed using validated methods (Appendix 4B) [32].

Postoperative data were grouped into four intervals: (i) ​< ​3 months; (ii) 3 months; (iii) 12 months; and (iv) ​> ​12 months after TKA. This classification was informed by evidence that knee pain decreases most within the first 3 months postoperatively and remains stable for at least one year [52], while walking speed fluctuates during the first 5 months, improves between 6 and 12 months, and slightly declines after 13 months [14].

Subgroup and overall pooled effect sizes were computed using the “metamean” function from the General Package for Meta-Analysis (version 7.0.0) in R (version 4.3.3) [48], with inverse variance weighting [49]. Heterogeneity was assessed using the I^2^ statistic (thresholds: < 25 ​% low, 25–50 ​% moderate, 50–75 ​% high, > 75 ​% very high) [53]. Forest plots displayed effect sizes and heterogeneity.

Meta-regression examined associations between walking speed and knee pain using univariate and multivariate models, based on study-level means and SDs. Sex was coded as the proportion of female participants, and pain measurement tool as a dichotomous variable (WOMAC-pain vs. KOOS-pain). Repeated measures were addressed by including both PRE and POST time points from each study. Meta-regression models were fitted using the Meta-Analysis Package (version 4.6.0) for R (version 4.3.3).

The univariate model used knee pain as the sole predictor for walking speed. The multivariate model adjusted for age, sex, BMI, time of measurement, and pain measurement tool. While evidence is mixed, some studies report negative associations between walking speed and older age [54], female sex [55], and higher BMI [55], whereas others report no associations [14]. Walking speed may also vary by postoperative time point due to recovery trajectory [14,15]. The pain measurement tool was included to account for differences between WOMAC-pain and KOOS-pain, with the latter containing additional items [34,36]. Predictors with p-values > 0.20 were removed sequentially using backward stepwise regression until only significant (p ​< ​0.05) predictors remained [56].

The final model was evaluated for key meta-regression assumptions. Linearity and heteroskedasticity were assessed using residuals versus fitted plots, and residual normality using the Shapiro-Wilk test and histogram inspection. Multicollinearity was checked using correlation matrices and variance inflation factors, with values ​> ​10 indicating collinearity [57]. Outliers and influential studies were scrutinized using difference in beta statistics (|DFBETAS| ​> ​2/√n) and absolute studentized residuals (|t| ​> ​2) [58]. Studies exceeding either threshold had both PRE and POST observations excluded. All tests were two-tailed (α ​= ​0.05).

## 3 Results

### 3.1 Study characteristics

**Fig. 1:**
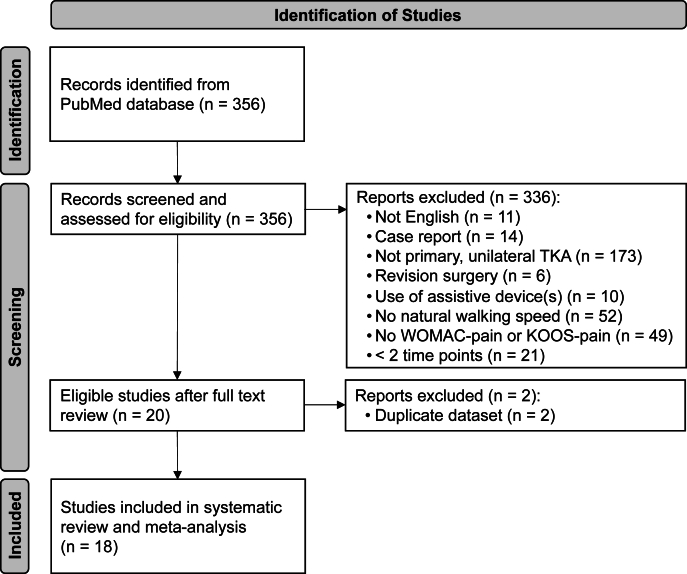
**PRISMA flowchart of study selection.** Flow diagram depicting the inclusion and exclusion process following the Preferred Reporting Items for Systematic Reviews and Meta-Analyses (PRISMA) guidelines.

The PubMed database search identified 356 studies, of which 20 met all inclusion criteria. After further review, two studies were excluded as duplicates of two other datasets reporting changes in knee pain and walking speed at 3 and 12 months post-TKA [59,60]. Consequently, only data from the earliest published studies were retained [61,62]. Screening the reference lists of identified review articles on knee pain and walking speed in TKA patients yielded no additional eligible studies. Ultimately, 18 studies were included in this systematic review and meta-analysis, comprising a total of 819 patients (64.1 ​% female; age ​= ​65.1 ​± ​8.3 years; BMI ​= ​28.4 ​± ​5.9 ​kg/m^2^) (Table 1; Fig. 1).

**Table 1:** Characteristics of included studies, including sample size, patient demographics, measurement methods for knee pain and walking speed, measurement time points, and PRE and POST values for knee pain and walking speed and their respective changes. Values are given as the frequency (*n*) or mean ​± ​standard deviation (SD).

Study	Sample size (female/male)	Age (years)	BMI (kg/m^2^)	Knee pain measurement method (scale)	Walking speed measurement method	Inclusion of control group	Time of measurement PRE (days)^a^	Time of measurement POST (months)^a^	Knee pain PRE (points) (original scale)	Knee pain POST (points) (original scale)	Knee pain PRE (points) (standardized scale)	Knee pain POST (points) (standardized scale)	Walking speed PRE (m/s)	Walking speed POST (m/s)	Knee pain change (points)^c^	Walking speed change (m/s)^c^	Reference
Abdel et al., 2014^b^	40 (24/16)	71.0 ​± ​6.0	29.2 ​± ​4.1	KOOS (0–100)	3D motion analysis system (Vicon, Oxford, UK)	No	N/A	3	22.5 ​± ​10.4	34.5 ​± ​11.9	77.5 ​± ​10.4	65.5 ​± ​11.9	0.64 ​± ​0.14	0.76 ​± ​0.16	−12.0 ​± ​14.9	0.12 ​± ​0.19	[67]
An et al., 2023^b^	40 (40/0)	70.8 ​± ​3.1	26.0 ​± ​3.1	WOMAC (0–20)	Pressure mapping system (Zebris PDM platform, FDM 1.5, Zebris Medical GmbH, Isny, Germany)	No	1	1.5	15.3 ​± ​2.6	5.5 ​± ​8.5	61.1 ​± ​9.4	27.4 ​± ​13.4	0.67 ​± ​0.15	0.74 ​± ​0.14	−33.8 ​± ​15.8	0.07 ​± ​0.07	[74]
Bączkowicz et al., 2018	21 (14/7)	63.5 ​± ​9.5	29.1 ​± ​4.6	WOMAC (0–20)	Pressure mapping system (GAITRite portable, CIR systems, Clifton, NJ, USA)	Yes	1	3	11.5 ​± ​6.2	5.9 ​± ​4.8	57.5 ​± ​31.0	29.5 ​± ​24.0	0.47 ​± ​0.15	0.58 ​± ​0.19	−28.0 ​± ​40.4	0.11 ​± ​0.17	[73]
Benner et al., 2024^b^	85 (45/40)	66.4 ​± ​8.0	28.2 ​± ​4.5	KOOS (0–100)	Stopwatch	No	N/A	3 12	43.3 ​± ​9.4	69.2 ​± ​18.5 84.7 ​± ​17.0	56.7 ​± ​9.4	30.8 ​± ​18.5 15.3 ​± ​17.0	1.15 ​± ​0.03	1.18 ​± ​0.25 1.34 ​± ​0.23	−25.9 ​± ​21.3 −41.4 ​± ​20.0	0.03 ​± ​0.24 0.18 ​± ​0.22	[70]
Berghmans et al., 2018	150 (79/71)	64.7 ​± ​8.1	30.9 ​± ​4.9	WOMAC (0–20)	Pressure mapping system (GAITRite system, CIR systems, Clifton, NJ, USA)	No	1	3 12	10.6 ​± ​4.1	16.0 ​± ​4.1 17.6 ​± ​4.4	47.0 ​± ​20.5	20.0 ​± ​20.5 12.0 ​± ​22.0	0.99 ​± ​0.23	1.08 ​± ​0.20 1.17 ​± ​0.20	−27.0 ​± ​29.9 −35.0 ​± ​31.0	0.09 ​± ​0.21 0.18 ​± ​0.21	[72]
Boekesteijn et al., 2022	24 (12/12)	63.0 ​± ​7.5	28.3 ​± ​4.3	KOOS (0–100)	Inertial sensors (Opal V2, APDM Inc., Portland, OR)	Yes	N/A	2	N/A	N/A	N/A	N/A	N/A	N/A	−28.0 ​± ​20.0	−0.04 ​± ​0.17	[63]
Bonnefoy-Mazure et al., 2017	71 (46/25)	68.1 ​± ​7.2	30.2 ​± ​5.5	WOMAC∗ (0–100)	3D motion analysis system (Vicon, Oxford, UK)	Yes	7	12	44.8 ​± ​17.3	82.5 ​± ​15.2	55.2 ​± ​17.3	17.5 ​± ​15.2	1.10 ​± ​0.20	1.30 ​± ​0.20	−37.7 ​± ​23.8	0.20 ​± ​0.19	[61]
Bonnefoy-Mazure et al., 2022	28 (17/11)	66.4 ​± ​12.2	31.7 ​± ​9.4	WOMAC∗ (0–100)	3D motion analysis system (Vicon, Oxford, UK)	No	7	84	40.6 ​± ​9.4	83.3 ​± ​20.8	59.4 ​± ​9.4	16.7 ​± ​20.8	1.04 ​± ​0.21	0.94 ​± ​0.20	−36.6 ​± ​27.8	−0.11 ​± ​0.30	[62]
Dabirrahmani et al., 2024	16 (9/7)	67.1 ​± ​6.8	32.2 ​± ​5.7	WOMAC (0–20)	3D motion analysis system (Vicon, Oxford, UK)	Yes	30–45	12	11.3 ​± ​3.0	2.8 ​± ​1.8	56.3 ​± ​14.9	10.9 ​± ​8.8	0.95 ​± ​0.20	1.12 ​± ​0.15	−45.4 ​± ​17.8	0.17 ​± ​0.18	[78]
Debbi et al., 2015	50 (28/22)	65.9 ​± ​8.1	33.5 ​± ​4.4	WOMAC (0–10)	3D motion analysis system (Vicon, Oxford, UK)	No	14	1.5	6.1 ​± ​2.3	5.8 ​± ​2.2	61.0 ​± ​23.0	58.0 ​± ​22.0	0.81 ​± ​0.18	0.73 ​± ​0.21	−3.0 ​± ​32.9	−0.08 ​± ​0.19	[76]
Kondo et al., 2024^b^	41 (34/7)	74.2 ​± ​5.6	27.2 ​± ​4.2	WOMAC (0–20)	Stopwatch	No	1	0.75 12	8.4 ​± ​3.8	6.5 ​± ​3.3 3.0 ​± ​3.0	42.2 ​± ​19.2	32.3 ​± ​16.7 15.1 ​± ​15.0	1.11 ​± ​0.32	1.05 ​± ​0.28 1.43 ​± ​0.30	−9.8 ​± ​26.6 −27.1 ​± ​25.6	−0.06 ​± ​0.30 0.32 ​± ​0.31	[75]
Naili et al., 2017	28 (18/10)	65.7 ​± ​7.3	29.6 ​± ​4.6	KOOS (0–100)	3D motion analysis system (Vicon, Oxford, UK)	Yes	30	12	45.3 ​± ​15.2	78.0 ​± ​20.6	54.7 ​± ​15.2	22.0 ​± ​20.6	1.11 ​± ​0.20	1.17 ​± ​0.18	−32.7 ​± ​22.0	0.07 ​± ​0.15	[77]
Ro et al., 2018	23 (23/0)	67.1 ​± ​5.7	26.8 ​± ​4.3	WOMAC (0–10)	3D motion analysis system (motion analysis, Santa Rosa, CA, USA)	No	N/A	24	4.7 ​± ​4.1	1.1 ​± ​1.3	47.0 ​± ​41.0	11.0 ​± ​13.0	0.88 ​± ​0.11	0.91 ​± ​0.10	−36.0 ​± ​45.5	0.03 ​± ​0.11	[64]
Tali et al., 2010	26 (26/0)	70.0 ​± ​6.5	32.4 ​± ​5.3	WOMAC (0–50)	Pressure mapping system (Footscan Scientific 3D Box, Rsscan International, Belgium)	Yes	1	3	24.2 ​± ​6.0	6.8 ​± ​5.2	48.3 ​± ​12.1	13.5 ​± ​10.3	0.76 ​± ​0.35	1.01 ​± ​0.34	−34.8 ​± ​16.4	0.25 ​± ​0.34	[71]
Turcot et al., 2013	78 (48/30)	68.0 ​± ​8.0	23.7 ​± ​8.0	WOMAC∗ (0–100)	3D motion analysis system (Vicon, Oxford, UK)	Yes	N/A	3	43.1 ​± ​17.5	69.6 ​± ​15.3	56.9 ​± ​17.5	30.4 ​± ​15.3	1.06 ​± ​0.21	1.09 ​± ​0.22	−26.5 ​± ​24.0	0.03 ​± ​0.21	[68]
Wegrzyn et al., 2013^b^	36 (28/8)	65.5 ​± ​7.6	30.5 ​± ​5.1	KOOS (0–100)	3D motion analysis system (motion analysis, Santa Rosa, CA, USA)	No	N/A	2	17.5 ​± ​5.0	28.5 ​± ​6.1	82.5 ​± ​5.0	71.5 ​± ​6.1	0.87 ​± ​0.20	1.00 ​± ​0.16	−11.0 ​± ​9.2	0.13 ​± ​0.14	[69]
Wellman et al., 2017^b^	40 (21/19)	62.6 ​± ​7.5	30.6 ​± ​5.1	KOOS (0–100)	Stopwatch	No	N/A	12	50.0 ​± ​15.7	92.4 ​± ​9.3	50.0 ​± ​15.7	7.6 ​± ​9.3	1.19 ​± ​0.25	1.35 ​± ​0.24	−42.4 ​± ​18.3	0.16 ​± ​0.23	[65]
Yaari et al., 2015	22 (13/9)	67.3 ​± ​8.4	28.3 ​± ​3.1	WOMAC (0–100)	Pressure mapping system (GaitMat system, E.Q. Inc., Chalfont, PA, USA)	No	N/A	3	39.2 ​± ​24.3	23.0 ​± ​23.2	39.2 ​± ​24.3	23.0 ​± ​23.2	0.71 ​± ​0.24	1.00 ​± ​0.21	−16.2 ​± ​26.1	0.29 ​± ​0.22	[66]

Note: N/A ​= ​not available; BMI ​= ​body mass index; PRE ​= ​preoperative (i.e., baseline measurement before TKA); POST ​= ​postoperative (i.e., follow-up measurement after TKA); WOMAC = Western Ontario and McMaster Universities Osteoarthritis Index pain subscale (0 ​= ​no pain, 100 ​= ​worst pain); WOMAC∗ ​= ​inverted WOMAC (0 ​= ​worst pain, 100 ​= ​no pain); KOOS = Knee Injury and Osteoarthritis Outcome Score pain subscale (0 ​= ​worst pain, 100 ​= ​no pain).

a SDs not reported for measurement time points.

b Combined values from two TKA patient groups.

c Change-from-baseline values were calculated as POST values minus PRE values.

All included studies examined pre-to-post changes in knee pain and walking speed, with POST measurements collected between 3 weeks and 84 months (7 years) after TKA. Regarding PRE measurements, eight studies did not specify the specific timing of assessments [[63], [64], [65], [66], [67], [68], [69], [70]]. Among those that reported a specific time point, five evaluated patients one day before TKA [[71], [72], [73], [74], [75]], two at seven days before TKA [61,62], one at 14 days before TKA [76], and two at 30 days before TKA [77,78]. Regarding POST measurements, five studies assessed patients within 3 months (i.e., 0.75–2 months) post-TKA [63,69,[74], [75], [76]]; seven studies at exactly 3 months [[66], [67], [68],[70], [71], [72], [73]]; seven studies at 12 months [61,65,70,72,75,77,78] (one of which also reported data within 3 months [75] and two at 3 months [70,72]); and two studies beyond 12 months (one at 24 months and one at 84 months) [62,64] (Table 1).

Six studies reported multiple TKA patient groups receiving different treatments, including different knee implant types [65], surgical procedures [67,69,70] or exercise interventions [74,75]; their summary statistics were combined in the analysis. Additionally, one study reported two closely spaced POST time points (3 and 3.7 months) [73]; only the 3-month data were retained to align with other included studies.

A variety of point scales and measurement methods were used to assess knee pain and walking speed across studies. Knee pain was measured using WOMAC-pain in 12 studies [61,62,64,66,68,[71], [72], [73], [74], [75], [76],^78^] and KOOS-pain in six studies [63,65,67,69,70,77] (Table 1). Among WOMAC-pain studies, five used a Likert scale [62,68,72,73,75], two used a visual analogue scale [71,76], and five did not specify the scale format [61,64,66,74,78]. The WOMAC-pain scoring ranges varied: four studies used a 0–100 scale [61,62,66,68], one a 0–50 scale [71], five a 0–20 scale [[72], [73], [74], [75],^78^], and two a 0–10 scale [64,76]; of these, three reported inverted WOMAC-pain scores [61,62,68]. In contrast, all KOOS-pain studies used a 0–100 Likert scale [63,65,67,69,70,77].

Walking speed was predominantly reported in m/s, except one study using kilometers per hour [74]. Measurement methods varied: nine studies used three-dimensional motion analysis systems [61,62,64,[67], [68], [69],[76], [77], [78]], five used pressure mapping systems [66,[71], [72], [73], [74]], one used inertial sensors [63], and three used a stopwatch [65,70,75].

### 3.2 Risk of bias

**Fig. 2:**
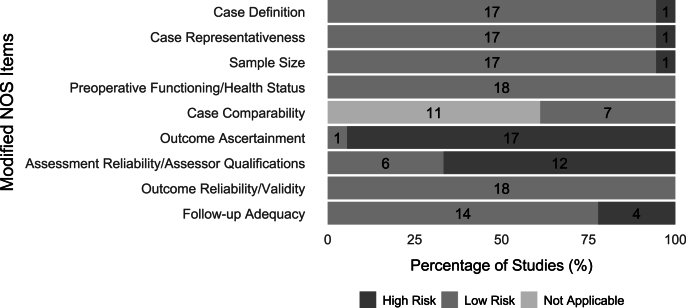
**Summary of risk of bias assessment.** The risk of bias for each study was evaluated using a modified Newcastle-Ottawa Scale (NOS). Studies were classified as having either low or high risk of bias based on predefined criteria across the following categories: selection bias (case definition, case representativeness, sample size), measurement bias (preoperative functioning/health status, case comparability), and observation bias (outcome ascertainment, assessment reliability/assessor qualifications, outcome reliability/validity, follow-up adequacy). Studies without a control group were marked as "Not Applicable" in the comparability assessment between cases and controls.

**Fig. 3:**
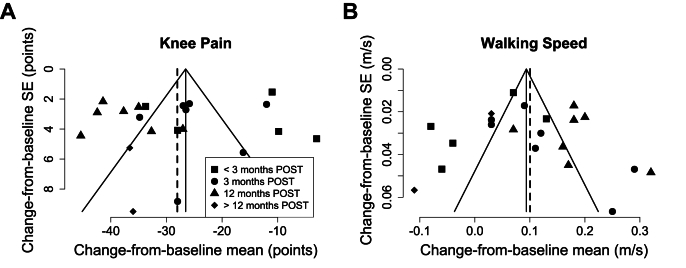
**Funnel plots of publication bias and study heterogeneity.** Funnel plots illustrate the risk of bias for (A) combined WOMAC-pain and KOOS-pain scores, and (B) walking speed. Data points represent individual studies, with symbols indicating the time point for change-from-baseline measurements: squares (< 3 months POST), circles (3 months POST), triangles (12 months POST), and rhombuses (> 12 months POST). In general, larger studies cluster at the top of the plot, while smaller studies exhibit greater variability and scatter at the bottom. In the absence of bias, the funnel plot should display a symmetrical, inverted funnel shape around the summary estimate, shown by a solid vertical line (common effect model) and a dashed vertical line (random effects model). Asymmetry may suggest publication bias and/or study design heterogeneity, indicating the need for additional studies to strengthen conclusions. SE: standard error of the mean.

The risk of bias was evaluated using a modified NOS scale. The percentage of studies categorized as low or high risk of bias for each NOS item is summarized in Fig. 2. All studies showed low risk of bias in preoperative functioning and health status, and reliability and validity of outcome measures. In contrast, one study (5.6 ​%) had a high risk of bias for case definition and case representativeness, as it did not report prior patient surgeries or confirm that only individuals with clinically diagnosed knee OA were included [71]. Another study (5.6 ​%) was at high risk due to a total sample size of fewer than 20 patients [78]. Furthermore, 17 studies (94.4 ​%) did not blind outcome assessors to measurement time points [[61], [62], [63], [64], [65], [66], [67], [68],[70], [71], [72], [73], [74], [75], [76], [77], [78]], 12 studies (66.7 ​%) lacked information on assessor qualifications and experience [[61], [62], [63],^65^,^67^,^68^,^70^,^71^,^73^,^75^,^76^,^78^], and four studies (22.2 ​%) had follow-up losses exceeding 15 ​% of the sample [61,62,73,77]. Despite these limitations, all studies were ultimately classified as having an overall low risk of bias. Funnel plots assessing publication bias and study design heterogeneity are presented in Fig. 3. The observed asymmetry, indicated by the absence of the expected funnel shape, suggests potential publication bias and/or heterogeneity in study designs.

### 3.3 Longitudinal changes in knee pain after TKA

**Fig. 4:**
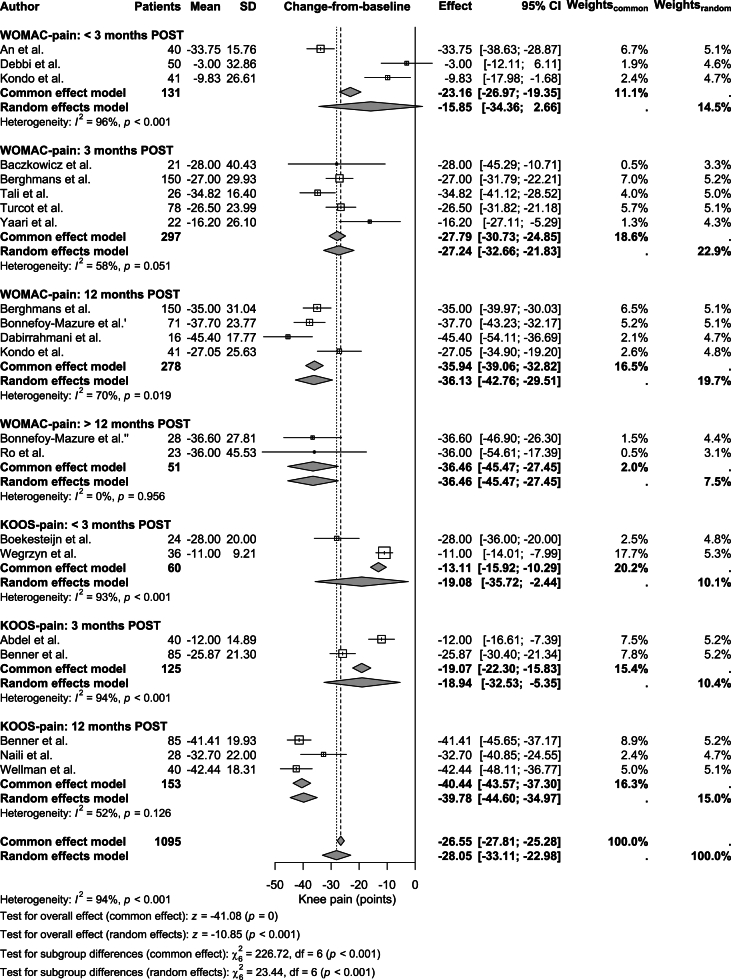
**Forest plot of change-from-baseline effects for knee pain.** The plot displays changes in WOMAC-pain and KOOS-pain scores at < 3 months, 3 months, 12 months, and > 12 months post-TKA. Box sizes for individual studies are proportional to their inverse-variance weights under the random effects model, reflecting the precision and relative contribution of each study. Pooled effects, reported as mean differences with 95 ​% confidence intervals (CIs), were calculated using a random effects model to account for between-study heterogeneity. The overall pooled change-from-baseline in WOMAC-pain was −28.4 points (95 ​% CI: −34.5 to −22.3, p ​< ​0.001; I^2^ ​= ​87 ​%), and in KOOS-pain was −27.5 points (95 ​% CI: −37.0 to −18.0, p ​< ​0.001; I^2^ ​= ​97 ​%) (values not shown in figure). WOMAC-pain: pain subscale of the Western Ontario and McMaster Universities Osteoarthritis Index; KOOS-pain: pain subscale of the Knee Injury and Osteoarthritis Outcome Score; PRE: preoperative; POST: postoperative.

Changes-from-baseline in WOMAC-pain scores were available for all four POST time points and generally indicated knee pain reduction, ranging from −45.4 to −3.0 points (Table 1; Fig. 4). At < 3 months POST, three studies showed a pooled (non-significant) reduction of −15.9 points (95 ​% CI: −34.4 to 2.7, p ​= ​0.093; I^2^ ​= ​96 ​%). At 3 months POST, five studies showed a pooled reduction of −27.2 points (95 ​% CI: −32.7 to −21.8, p ​< ​0.001; I^2^ ​= ​58 ​%). At 12 months POST, four studies showed a pooled reduction of −36.1 points (95 ​% CI: −42.8 to −29.5, p ​< ​0.001; I^2^ ​= ​70 ​%). Finally, at > 12 months POST, two studies showed a pooled reduction of −36.5 points (95 ​% CI: −45.5 to −27.4, p ​< ​0.001; I^2^ ​= ​0 ​%). The overall pooled change-from-baseline in WOMAC-pain was −28.4 points (95 ​% CI: −34.5 to −22.3, p ​< ​0.001; I^2^ ​= ​87 ​%).

Changes-from-baseline in KOOS-pain were available for three of the four POST time points, showing consistent knee pain reductions ranging from −42.4 to −11.0 points (Table 1; Fig. 4). At < 3 months POST, two studies showed a pooled reduction of −19.1 points (95 ​% CI: −35.7 to −2.4, p ​= ​0.025; I^2^ ​= ​93 ​%). At 3 months POST, two studies reported a reduction of −18.9 points (95 ​% CI: −32.5 to −5.4, p ​= ​0.006; I^2^ ​= ​94 ​%). At 12 months POST, three studies showed a pooled reduction of −39.8 points (95 ​% CI: −44.6 to −35.0, p ​< ​0.001; I^2^ ​= ​52 ​%). No studies reported KOOS-pain values beyond 12 months. The overall pooled change-from-baseline in KOOS-pain was −27.5 points (95 ​% CI: −37.0 to −18.0, p ​< ​0.001; I^2^ ​= ​97 ​%). Combining WOMAC-pain and KOOS-pain scores, the overall pooled change-from-baseline in knee pain was −28.1 points (95 ​% CI: −33.1 to −23.0, p ​< ​0.001; I^2^ ​= ​94 ​%).

### 3.4 Longitudinal changes in walking speed after TKA

**Fig. 5:**
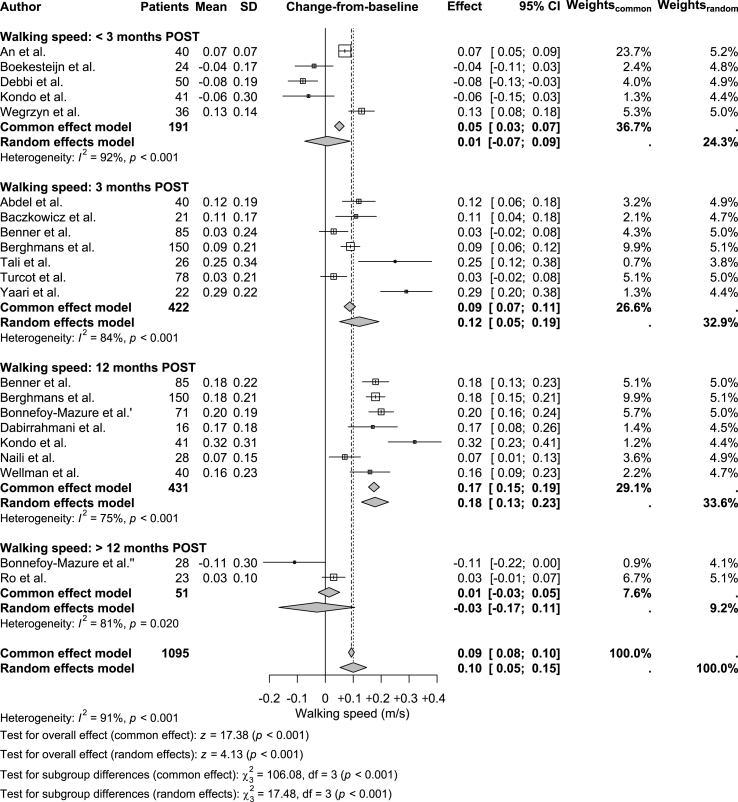
**Forest plot of change-from-baseline effects for self-selected walking speed.** The plot displays changes in self-selected walking speed at < 3 months, 3 months, 12 months, and > 12 months post-TKA. Box sizes for individual studies are proportional to their inverse-variance weights under the random effects model, reflecting the precision and relative contribution of each study. Pooled effects, reported as mean differences with 95 ​% confidence intervals (CIs), were calculated using a random effects model to account for between-study heterogeneity. PRE: preoperative; POST: postoperative.

Changes-from-baseline in walking speed were available for all four POST time points. Unlike the consistent reductions observed in knee pain, changes in walking speed varied across time points, ranging from −0.11 ​m/s to +0.32 ​m/s (Table 1; Fig. 5). At < 3 months POST, five studies showed a pooled (non-significant) increase of +0.01 ​m/s (95 ​% CI: −0.07 to 0.09, p ​= ​0.848; I^2^ ​= ​92 ​%). At 3 months POST, seven studies showed a pooled increase of +0.12 ​m/s (95 ​% CI: 0.05 to 0.19, p ​< ​0.001; I^2^ ​= ​84 ​%). At 12 months POST, seven studies showed a pooled increase of +0.18 ​m/s (95 ​% CI: 0.13 to 0.23, p ​< ​0.001; I^2^ ​= ​75 ​%). Finally, at > 12 months POST, two studies showed a pooled (non-significant) reduction of −0.03 ​m/s (95 ​% CI: −0.17 to 0.11, p ​= ​0.664; I^2^ ​= ​81 ​%; Fig. 4). The overall pooled change-from-baseline in walking speed was +0.10 ​m/s (95 ​% CI: 0.05 to 0.15, p ​< ​0.001; I^2^ ​= ​91 ​%).

### 3.5 Association between knee pain and walking speed

Of the 18 studies included in the systematic review and meta-analysis, 17 were eligible for the meta-regression analysis, contributing a total of 37 observations (17 PRE, 20 POST). One study was excluded because it reported only change-from-baseline values [63], while three others contributed two POST time points (e.g., 3 and 12 months) [70,72,75].

**Fig. 6:**
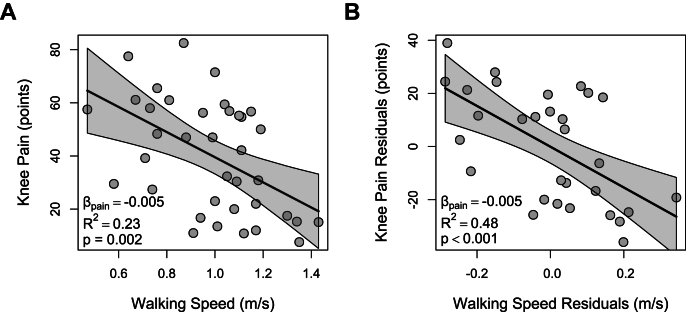
**Meta-regression analyses of the association between walking speed and knee pain.** (A) Univariate meta-regression showing that higher knee pain is significantly associated with slower walking speed across pre- and post-TKA time points. (B) Multivariate partial regression plot depicting the negative association between knee pain and walking speed, adjusted for pain measurement tool (WOMAC-pain vs. KOOS-pain). Each plot displays individual study-level observations (grey dots), the fitted regression line (black diagonal), and the 95 ​% confidence interval (shaded area). Regression coefficients, R^2^, and p-values are provided for both models. Analyses were based on 37 observations from 17 studies.

In the univariate meta-regression, knee pain was a significant independent predictor of walking speed (β ​= ​−0.005, R^2^ ​= ​0.23, p ​= ​0.002), indicating that higher pain was associated with slower walking speed (Fig. 6A).

The final multivariate meta-regression model, identified through backward stepwise elimination, retained knee pain and pain measurement tool as significant predictors (p ​< ​0.05). Model diagnostics revealed violations of linearity and homoskedasticity, along with three influential outliers across PRE and POST time points. These studies had DFBETAS values for knee pain (|DFBETAS| ​= ​0.36–0.39), exceeding the threshold of 0.33 [67,73,75], with two also showing studentized residuals (|t| ​= ​2.02–2.50) above the threshold of 2 [73,75]. After excluding these outliers, the refined model satisfied all assumptions. Knee pain (β ​= ​−0.005, p ​< ​0.001) and use of WOMAC-pain (vs. KOOS-pain) (β ​= ​−0.224, p ​< ​0.001) remained significant predictors, each independently and negatively associated with walking speed. The model explained a substantial proportion of the variance in walking speed (R^2^ ​= ​0.48, p ​< ​0.001) (Fig. 6B). The model also showed significant residual heterogeneity (I^2^ ​= ​96 ​%), suggesting additional unexplained factors influencing this relationship.

## 4 Discussion

This systematic review and meta-analysis is the first to examine simultaneous changes in knee pain and walking speed following primary, unilateral TKA for knee OA, and to explore their relationship. Across 18 included studies with overall low risk of bias, we found substantial postoperative pain relief and clinically meaningful – but time-dependent – walking speed gains. Statistically significant pain reductions were evident by ​< ​3 months (KOOS-pain) and 3 months (WOMAC-pain), with sustained improvements up to and beyond 12 months. In contrast, walking speed improved significantly only at 3 and 12 months, with no early postoperative change and a non-significant decline beyond one year. These findings highlight the partly independent recovery trajectories of pain and mobility after TKA and underscore the importance of addressing both domains in postoperative care.

The magnitude of pain relief observed after TKA was substantial and exceeded established clinical thresholds. Pooled WOMAC-pain reductions of 27–36 points (at 3, 12, and > 12 months) and KOOS-pain reductions of 19–40 points (at ​< ​3 and 12 months) were greater than minimal clinically important differences (MCIDs) of 11–28 points and minimum detectable change with 95 ​% confidence (MDC95) values of 22–23 points for WOMAC-pain [79,80]. These results are broadly consistent with earlier reviews reporting postoperative pain improvement [4,11,12], but extend the literature by quantifying absolute pre-to-post changes in validated pain and function outcomes. Prior reviews, however, had important methodological limitations. Beswick et al. (2012) did not account for preoperative knee pain levels, preventing quantification of change, and reported postoperative pain only as categorical outcomes (‘favourable’, ‘unfavourable’, or ‘uncertain’) rather than continuous scores [11]. Shan et al. (2015) based their meta-analysis of pain outcomes on only five studies, some of which included mixed unilateral and bilateral surgeries [4]. Sayah et al. (2021) described the clinical course of pain after primary TKA, showing that patients, on average, experienced marked improvements within the first 12 postoperative months, with minimal gains thereafter [12]. However, their analysis did not quantify absolute pre-to-postoperative reductions or assess whether these met clinical thresholds, and their meta-regression did not adjust for covariates such as age, sex, or BMI. By contrast, our analysis quantified absolute pain reductions against established thresholds and incorporated meta-regression with key covariates, thereby enhancing clinical interpretability and providing a more explanatory framework for between-study variability.

Our pooled walking speed gains (+0.10 ​m/s overall; +0.12 ​m/s at 3 months; +0.18 ​m/s at 12 months) align with prior reports (0.08–0.22 ​m/s) [14,15,43] and exceeded the +0.1 ​m/s threshold for meaningful change linked to better mobility and reduced health risk [21]. However, speed improvements were absent early postoperatively (perhaps due to acute postsurgical pain, inflammation, and limited weightbearing or mobility [81,82]) and not sustained beyond one year, supporting the need for longer-term rehabilitation strategies. Unlike prior reviews that included broader mixed surgical populations and protocols [14,15,43], our analysis focused exclusively on primary, unilateral TKA and self-selected walking speeds, enhancing applicability. Notably, walking speed remains impaired post-TKA relative to healthy peers [42,83], reinforcing the value of functional rehabilitation and positioning TKA as a means to restore mobility and promote timely intervention.

Meta-regression identified knee pain and the pain measurement tool (WOMAC-pain vs. KOOS-pain) as independent predictors of walking speed, together explaining 48 ​% of the variance. Age, sex, and BMI were not retained, likely reflecting limited variability across included studies and modest sample sizes and suggesting that pain burden may be a stronger determinant of mobility (walking performance) than demographic traits in this population. KOOS-pain, which includes function-specific items (e.g., pain during twisting, bending), may better reflect limitations relevant to gait than WOMAC-pain [36], warranting consideration when interpreting pain–function relationships.

Mechanical, rehabilitation, and pharmacological factors not accounted for in analyses likely contributed to the observed variability. In particular, postoperative pain medication use [[84], [85], [86]] – which was rarely reported in the included studies – may have affected reported pain and walking speed outcomes and contributed to heterogeneity. Other factors such as implant type [87,88], surgical approach [89,90], and rehabilitation practices [91,92] also varied widely across studies and may have influenced mobility outcomes and knee pain recovery. Quadriceps and hamstring weakness commonly persist post-TKA and can limit walking speed [28,54,93], while surveys highlight inconsistencies in post-TKA therapy delivery, frequency, and content [91,92,94]. Although joint mechanics often improve after TKA [95,96] – contributing to faster walking speeds [93,97] – persistent gait abnormalities may increase joint stress and anterior knee pain [[98], [99], [100]]. Knee pain may in turn disrupt walking mechanics [101], creating a bidirectional feedback loop that hinders optimal recovery.

These findings highlight the need for comprehensive postoperative care that combines pain relief with targeted functional rehabilitation – addressing neuromuscular function and gait mechanics. Pain reduction can alleviate disability and enhance quality of life [102], while walking speed gains are linked to broad health benefits across the lifespan [17,[21], [22], [23]]. Even modest improvements (+0.1 ​m/s) are associated with increased survival and reduced morbidity [21], and speeds above 1 ​m/s correlate with lower risks of disability, hospitalization, and mortality in older adults [103,104]. As a simple, scalable metric, walking speed can be monitored in both clinical and real-world settings using wearable technologies such as smartwatches and smartphones [105], and its integration into postoperative care may help identify patients at risk of persistent mobility limitations and inform tailored rehabilitation strategies.

This review has several limitations. Key clinical variables (e.g., prior surgeries, implant type, rehabilitation protocols, analgesic use) were inconsistently reported, limiting explanatory power. Inconsistencies in outcome measurement (e.g., pain scale formats, speed assessment methods) further constrained interpretability. These issues likely contributed to the high between-study heterogeneity (I^2^ ​> ​90 ​%) and residual heterogeneity in meta-regression (I^2^ ​= ​96 ​%), indicating the influence of unmeasured factors. Funnel plot asymmetry and the presence of small studies suggest potential small-study effects. Only two studies reported outcomes beyond 12 months, limiting insight into long-term recovery. Finally, most data reflected patients in their sixties undergoing primary, unilateral TKA, which may restrict generalizability to younger, bilateral or revision cases. Nevertheless, this review has several notable strengths. These include rigorous eligibility criteria, use of standardized outcomes, and a meta-regression analysis that adjusted for key covariates. In addition, the included studies encompassed diverse implant types, surgical approaches, and postoperative care protocols, which enhance the generalizability of our findings across different TKA contexts.

Future work should adopt harmonized outcomes and clearly report surgical and rehabilitation protocols to enable subgroup comparisons. Longitudinal studies beyond one year are needed to evaluate sustained recovery. Individual patient data meta-analyses could improve adjustment for key covariates and clarify the influence of patient-level factors on recovery trajectories.

In conclusion, primary, unilateral TKA provides substantial knee pain relief and moderate, time-sensitive improvements in walking speed. While pain improvements appear robust, functional gains may diminish over time. These findings support integrated postoperative care strategies that target both symptoms and mobility improvements to optimize long-term recovery and health.

## Contributions

NF, MS and NMB conceived and designed the research. NF and MS collected and assembled the data. MS and NMB performed the statistical analysis. NF, MS, GND, and NMB contributed to drafting the manuscript and to the interpretation and discussion of the results. All authors critically revised the article for important intellectual content, approved the final version of the manuscript, and agree to be accountable for all aspects of the work.

## Role of the funding source

This work was supported by funding from the German Research Foundation (DFG – Deutsche Forschungsgemeinschaft, Project ID 427826188 - SFB 1444/CRC 1444 (GND); BR 6698/1-1 (NMB)). In addition, travel funds were received from the German Society for Orthopaedic and Trauma Surgery (DGOU – Deutsche Gesellschaft für Orthopädie und Unfallchirurgie (NF)). The funding sources had no role in the design of this study; in the collection, analysis and interpretation of data; in the writing of the article; and in the decision to submit the article for publication.

## Declaration of competing interests

NF, MS, GND and NMB have no conflicts of interest to declare.
