# Supplementary material for: Changes in knee pain and walking speed following primary, unilateral total knee arthroplasty and their association: A systematic review and meta-analysis
Source: Osteoarthr Cartil Open. 2025 Oct 10;7(4):100694. doi: 10.1016/j.ocarto.2025.100694 (PMC12554042; doi:10.1016/j.ocarto.2025.100694)
Supplement: Multimedia component 1 [file mmc1.pdf]

Appendix 1. Medical Subject Headings (MeSH) Terms and Keywords

("gait speed"[All Fields] OR "gait velocity"[All Fields] OR "walking speed"[All Fields] OR "walking velocity"[All Fields] OR "gait"[MeSH Terms]) AND ("total knee arthroplasty"[All Fields] OR "TKA"[All Fields] OR "total knee replacement"[All Fields] OR "TKR"[All Fields] OR "arthroplasty"[MeSH Terms]) AND ("pain"[All Fields] OR "pain"[MeSH Terms] OR "Knee Injury and Osteoarthritis Outcome Score"[All Fields] OR "KOOS"[All Fields] OR "Western Ontario and McMaster Universities Osteoarthritis Index"[All Fields] OR "WOMAC"[All Fields]) AND "knee"[All Fields] AND 1000/01/01:2025/09/30[Date - Create]
