# Supplementary material for: Changes in knee pain and walking speed following primary, unilateral total knee arthroplasty and their association: A systematic review and meta-analysis
Source: Osteoarthr Cartil Open. 2025 Oct 10;7(4):100694. doi: 10.1016/j.ocarto.2025.100694 (PMC12554042; doi:10.1016/j.ocarto.2025.100694)
Supplement: Multimedia component 2 [file mmc2.pdf]

Appendix 2. Decision Tree for Study Inclusion

1. Written in English
2. Not a case report
3. Study accessible
4. Patients with primary, unilateral TKA
5. No other surgeries in addition to TKA or revision surgeries
6. No use of assistive devices or walking aids during walking speed assessments
7. Self-selected (natural/preferred) walking speed
8. Knee pain measured with WOMAC-pain or KOOS-pain
9. Knee pain and walking speed values reported for at least two time points, one pre-TKA and one post-TKA

Appendix 2. Decision tree for study inclusion in the systematic review and meta-analysis. This flowchart illustrates the study selection process, detailing the sequential inclusion criteria applied during screening. A "yes" response was required to proceed to the next step. TKA = total knee arthroplasty; WOMAC-pain = pain subscale of the Western Ontario and McMaster Universities Osteoarthritis Index; KOOS-pain = pain subscale of the Knee injury and Osteoarthritis Outcome Score.
