# Supplementary material for: Changes in knee pain and walking speed following primary, unilateral total knee arthroplasty and their association: A systematic review and meta-analysis
Source: Osteoarthr Cartil Open. 2025 Oct 10;7(4):100694. doi: 10.1016/j.ocarto.2025.100694 (PMC12554042; doi:10.1016/j.ocarto.2025.100694)
Supplement: Multimedia component 3 [file mmc3.pdf]

Appendix 3. Modified Newcastle-Ottawa Scale (NOS)

Selection Bias

Case Definition

Low risk of bias: Cases are clearly defined with inclusion/exclusion criteria based on clinical standards, such as:

- History of unilateral TKA and prior surgeries.
- Knee pain measured using validated scales (e.g., WOMAC-pain, KOOS-pain).
- Inclusion/exclusion criteria clearly described for any control group, if used.

High risk of bias: Cases are not clearly defined, or minimal criteria are used for inclusion/exclusion.

Case Representativeness

Low risk of bias: Cases are truly representative of the population with knee OA and sourced from the general population, such as:

- Patients with clinically confirmed knee OA undergoing TKA.
- Controls (if used) without a history of knee OA or surgery.

High risk of bias: Cases not representative of the general population, or there is evidence of selection bias.

Sample Size

Low risk of bias: The study includes 20 or more participants in total.

High risk of bias: The study includes fewer than 20 participants in total.

Measurement Bias

Preoperative Functioning/Health Status

Low risk of bias: Preoperative physical functioning or health status is appropriately controlled, such as:

- Use of standardized assessments for knee pain or walking speed before TKA.
- Assessment of baseline physical activity levels or comorbidities.

High risk of bias: Preoperative functioning or health status is not controlled or not reported.

Case Comparability

Low risk of bias: Cases are matched with controls for at least one confounder or statistical analyses adjust for potential confounders, such as:

- Age, sex, body mass index.

High risk of bias: Confounders are not controlled for in the design or analysis, or are not acknowledged.

Observation Bias

Outcome Ascertainment

Low risk of bias: Outcome assessors are blinded to pre/post-TKA status or case/control grouping.

High risk of bias: Outcome assessors are not blinded, or blinding status is unclear/not reported.

Assessment Reliability/Assessor Qualifications

Low risk of bias: Assessor qualifications and experience are described, and assessment reliability is established (e.g., using trained physiotherapists or validated tools).

High risk of bias: Assessor qualifications and experience are not reported, and reliability is not established.

Outcome Reliability/Validity

Low risk of bias: Outcome measures and measurement tools are clearly described, with referenced or demonstrated reliability/validity.

High risk of bias: Outcome measures or measurement tools are not adequately described, or their reliability/validity is not addressed.

Follow-up Adequacy

Low risk of bias: Follow-up is clearly reported, with minimal loss (< 15%), and characteristics of those lost to follow-up are described.

High risk of bias: Follow-up loss is not reported, or characteristics of dropouts are not described.
