# Supplementary material for: Changes in knee pain and walking speed following primary, unilateral total knee arthroplasty and their association: A systematic review and meta-analysis
Source: Osteoarthr Cartil Open. 2025 Oct 10;7(4):100694. doi: 10.1016/j.ocarto.2025.100694 (PMC12554042; doi:10.1016/j.ocarto.2025.100694)
Supplement: Multimedia component 4 [file mmc4.pdf]

Appendix 4. Statistical Estimation and Imputation Methods

A) Pooling of Patient Demographics

The pooled mean was calculated as:

$$\bar{X}_{pooled} = \frac{\sum_{i=1}^{k}(X_i \times n_i)}{\sum_{i=1}^{k} n_i}$$

where $\bar{X}_{pooled}$ is the pooled weighted mean, k is the total number of studies, X_i is the mean of the i-th study, and n_i is the sample size of the i-th study.

The pooled standard deviation (SD), accounting for both within- and between-study variability, was calculated as:

$$\overline{SD}_{pooled} = \sqrt{\frac{\sum_{i=1}^{k}((n_i - 1) \times \left(SD_i^2 + \left(X_i - \bar{X}_{pooled}\right)^2\right))}{\sum_{i=1}^{k}(n_i - 1)}}$$

where $\overline{SD}_{pooled}$ is the pooled weighted SD, k is the total number of studies, SD_i is the SD of the i-th study, X_i is the mean of the i-th study, $\bar{X}_{pooled}$ is the overall weighted mean, and n_i is the sample size of the i-th study.

B) Estimating Missing Change-from-Baseline SDs

In studies reporting SDs at each time point along with a paired t-test statistic, the change-from-baseline SD was calculated as:

$$SD_{Change} = \left|\frac{MD}{t}\right| \times \sqrt{n}$$

where SD_{Change} is the change-from-baseline SD, MD is the mean difference between PRE and POST values, t is the t-statistic for the difference between PRE and POST values, and n is the number of participants.

For studies that did not report a paired t-statistic, a correlation coefficient (CC) was required to impute the change-from-baseline SD. When sufficient data were available, CCs were calculated using the following formula:

$$CC = \frac{SD_{PRE}^2 + SD_{POST}^2 - SD_{Change}^2}{2 \times SD_{PRE} \times SD_{POST}}$$

where CC is the correlation coefficient, SD_{PRE} is the SD at the pre-TKA time point, SD_{POST} is the SD at the post-TKA time point, and SD_{Change} is the change-from-baseline SD.

To minimize the impact of small-sample studies on the overall CC, a sample size-weighted average CC was calculated separately for knee pain and walking speed. The resulting weighted CCs were 0 for knee pain and 0.5 for walking speed, although individual study CCs varied. To assess the robustness of these imputations, sensitivity analyses were performed by varying the CC from 0 to 1 in 0.1 increments. These analyses showed no apparent influence on pooled estimates in either the subgroup or overall analyses. Thus, the original weighted CCs were retained and used to impute missing change-from-baseline SDs using the following formula:

$$SD_{Change} = \sqrt{SD_{PRE}^2 + SD_{POST}^2 - (2 \times CC \times SD_{PRE} \times SD_{POST})}$$

where SD_{Change} is the change-from-baseline SD, SD_{PRE} and SD_{POST} are the SDs at the pre-TKA and post-TKA time points, respectively, and CC is the correlation coefficient.
